# Turbulent Drag Reduction Research on Biomimetic Surfaces Based on the Microstructural Characteristics of Shark Skin

**DOI:** 10.3390/biomimetics11080573

**Published:** 2026-08-11

**Authors:** Meihong Gao, Zhenjiang Wei, Zhengyang Wu, Chengchun Zhang, Chun Shen

**Affiliations:** 1 Department of Mechanical and Electronic Engineering, Heze University, Heze 274015, China; gaomeihong@hezeu.edu.cn (M.G.); zhenjiangwei@hezeu.edu.cn (Z.W.); 2 Weihai Institute of Bionics, Jilin University, Weihai 264400, China; zywu24@henau.edu.cn; 3 College of Mechanical & Electrical Engineering, Henan Agricultural University, Zhengzhou 450002, China; 4 National Key Laboratory of Automotive Chassis Integration and Bionics, Jilin University, Changchun 130022, China; shench@jlu.edu.cn

**Keywords:** turbulent drag reduction, biomimetic surfaces, shark skin, V-shaped grooves

## Abstract

In engineering fields such as aviation, shipping, and high-speed rail, system operational efficiency and energy consumption are largely determined by turbulent drag. The drag reduction design of shark skin-inspired micro-groove structures have opened up new avenues for improving aerodynamic efficiency, optimizing flow field characteristics, and saving energy, representing a highly promising research hotspot in the field of functional micro-structured surfaces. Addressing the issue of drag reduction for V-shaped grooves (frictional Reynolds number 85–695), this paper employs Design of Experiments (DOE) combined with high-precision numerical simulation to clarify the influence of groove height (*h*), groove width (*s*), and inflow velocity (*U*) on the drag reduction rate for bionic microgroove surfaces. The drag reduction mechanism is further revealed through the analysis of vorticity distribution, vortex core position, boundary layer velocity distributions, pulsating velocity fields, and Reynolds stress distributions. When the dimensionless height *h^+^* and width *s^+^* range from 8.50 to 29.75, these grooves can effectively reduce resistance. A maximum drag reduction rate of 12.33% is achieved at *h^+^* = *s^+^* = 25.29 and a flow velocity of 80.7 m/s (frictional Reynolds number 599). At low flow velocities, larger groove dimensions are favorable for drag reduction. In contrast, smaller groove dimensions are required under medium-to-high flow velocity conditions. The optimal microstructural dimensions of V-shaped grooves decrease as the inflow velocity increases. V-shaped grooves can lift turbulent vortex coherent structures, reduce pulsating velocities in the streamwise, normal, and spanwise directions, and decrease the peak values of Reynolds stress in the near-wall region. The results can provide a quantitative basis for the design and engineering applications of biomimetic riblet drag-reducing surfaces.

## 1. Introduction

Turbulent frictional resistance constitutes a predominant source of energy dissipation in numerous engineering applications, including aviation, maritime transportation, and high-speed rail systems [1,2,3,4]. In particular, the skin friction resistance on submerged or aerodynamic surfaces accounts for a substantial fraction of the total resistance. often exceeding 50% for marine vessels and up to 80% for underwater vehicles and high-speed trains [5]. Consequently, even marginal reductions in surface friction can yield significant improvements in energy efficiency, operational range, and velocity. This imperative has driven sustained interest in the development of practical drag reduction technologies.

Among the diverse array of the drag reduction strategies, passive flow control methods that require no external energy input or auxiliary devices have attracted considerable interest [6,7,8]. Notably, biomimetic non-smooth surfaces inspired by the dermal denticles of fast-swimming sharks have demonstrated remarkable potential [9]. The skin of sharks, such as the shortfin mako shark (*Isurus oxyrinchus*), is covered by placoid scales featuring streamwise microgrooves, which are known to modulate near-wall turbulent coherent structures and attenuate viscous drag under turbulent flow conditions [10,11]. Since the pioneering work of the NASA Langley Research Center in the 1970s, V-shaped grooves have been extensively investigated and validated as an effective passive drag reduction method [12]. The grooves suppress the movement of transverse turbulent vortices near the wall [13], effectively reducing the number of low-speed streaks and inhibiting the formation of hairpin vortices, arc vortices, and horseshoe vortices [14,15]. The maximum static pressure efficiency of a bionic fan with micro-grooves increased by 3.46% [16]. Kim et al. [17] employed micron-scale V-shaped streamlined structures to focus on hydrodynamic drag using the SUBOFF model, and the results showed that the maximum total drag reduction was 3%. Wong et al. [18] introduced a viscous vortex model to predict the drag reduction of riblets up to the optimal size, thereby eliminating the need for expensive direct numerical simulations (DNS) or experiments. The effect of riblets on the mean and turbulent statistics of a zero-pressure-gradient boundary layer was investigated [19]. The results indicate that the viscous drag of the riblets in the turbulent regime is reduced by 4–6% when compared to a smooth surface, and the efficacy of sharp V-shaped riblets is shown to be marginally higher than that of the curved riblets. Micro-riblets were installed on a wing with the NACA 2412 airfoil, and the three-riblet model achieved a drag reduction rate of 23% at an angle of attack of 16° [20].

Despite extensive CFD numerical optimization and experimental investigations on V-shaped riblet drag reduction [21,22,23,24], several key research issues remain to be addressed. Nearly all existing riblet optimization simulations are limited to a single fixed inflow velocity, failing to establish a quantitative scaling relationship between optimal riblet size and variable flow speed, which restricts their engineering application across low-to-high speed scenarios. Moreover, the mechanisms by which V-shaped grooves influence the turbulent boundary layer by modifying the average velocity distribution, pulsating velocity, and Reynolds stress under different flow conditions remain to be further elucidated. In this work, a numerical simulation scheme for turbulent channel flow over V-shaped groove surfaces is established based on the open-source computational fluid dynamics platform OpenFOAM. To accelerate the development of turbulence, two rows of staggered cubic columns are arranged at the inlet of the computational domain. A systematic DOE method is employed to investigate the functional relationship among *h*, *s*, *U*, and DR. The drag reduction mechanism is revealed by analyzing the vorticity distribution, vortex core position, boundary layer velocity distributions, pulsating velocity fields, and Reynolds stress. The results provide quantitative insights into the optimal geometric and flow parameters for drag reduction and contribute to the rational design of biomimetic surfaces for engineering applications.

## 2. Methods

### 2.1. Design of Computational Schemes

The DOE method is used to investigate the effects of the height *h*, width *s*, and inflow velocity *U* on the drag reduction rate of the V-shaped groove wall. Table 1 shows that the factor levels and codes for a second-order orthogonal rotation design. The ranges for the height and width of the grooves are both 0.05–0.3 mm, and the inflow velocity ranges from 5–100 m/s (frictional Reynolds number 85–695). The numerical calculation scheme is determined based on a ternary quadratic orthogonal rotation combination design. The number of factors in the computational model is *p* = 3. The total number of calculations (*N*) can be expressed by the following formula.

(1) $$N=m_{c}+m_{0}+{\;m}_{r}$$

(2) $$m_{c}={\;2}^{p}$$

(3) $$m_{0}=4\left(1+m_{c}^{1/2}\right)-2p$$

(4) $$m_{r}=2p$$

(5) $$r=m_{c}^{1/4}$$

(6) Reτ= δuτν

(7) $$\delta =0.381\frac{x}{\sqrt[{5}]{Re_{x}}}$$

(8) Rex =ρ U xμ

$m_{c}$ is the total number of calculation points for each factor with two levels (+1, −1). $m_{0}$ is the number of calculations when all factors are set to zero level. $m_{r}$ is the number of calculation points represented by asterisks distributed across p coordinate axes. *r* is the distance from the calculation point to the center point. According to the above formula, $m_{c}\;=\;8$,$\;m_{0}\;=\;9,{\;m}_{r}\;=\;6$, *r* = 1.682, and *N* = 23. ${Re}_{\tau }$ denotes the frictional Reynolds number and δ represents the thickness of the turbulent boundary layer. $u_{\tau }$ is the friction velocity near the wall. *ν* represents the kinematic viscosity of air. *Re_x_* is the local Reynolds number. *ρ* is the density of air. *U* denotes the free-stream velocity of air. *x* is the distance from the inlet. *μ* is the dynamic viscosity.

### 2.2. Design of the Computational Domain

**Figure 1:**
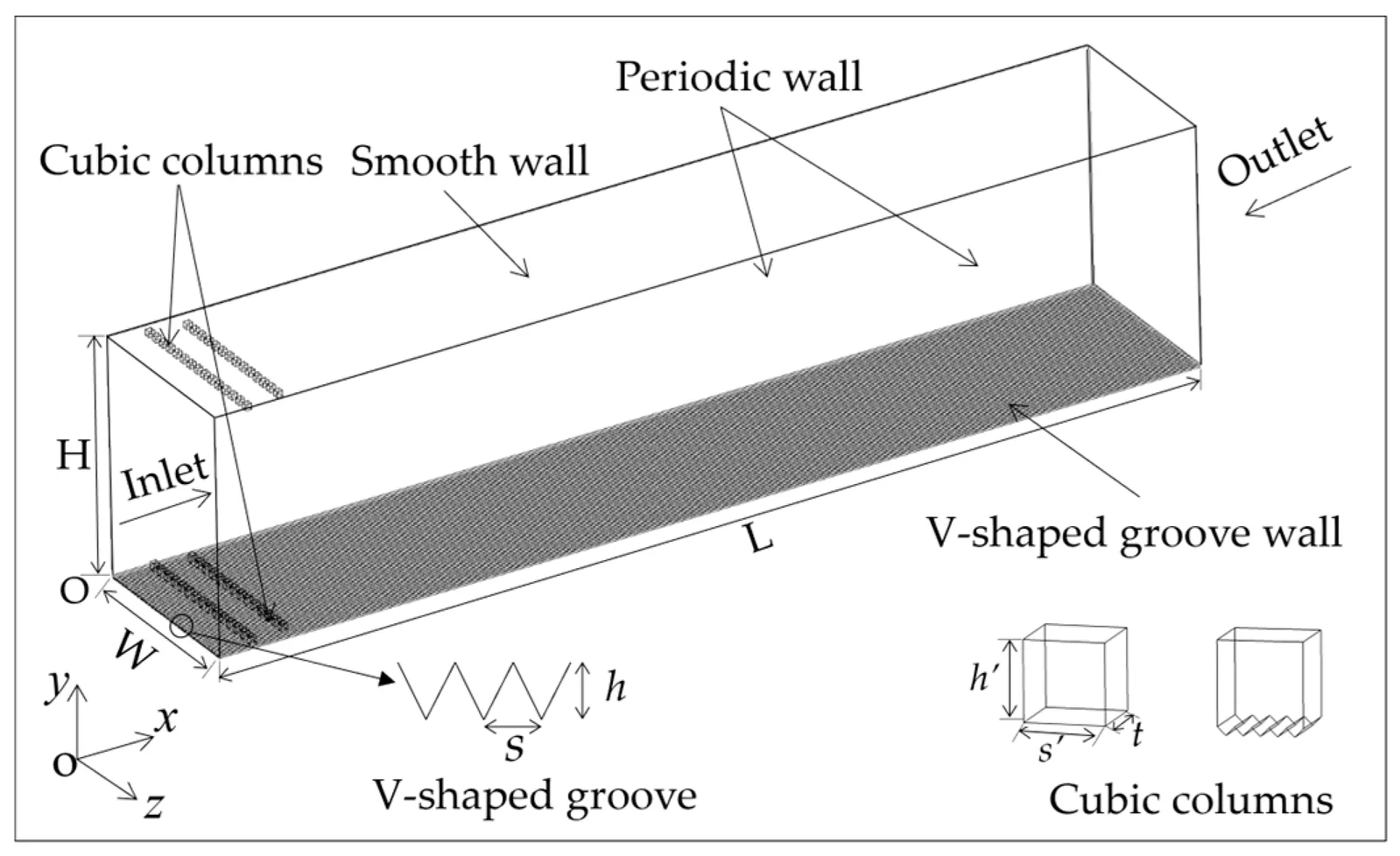
Computational domain.

Figure 1 shows that the upper wall of the computational domain is a smooth plate, and the lower wall features a V-shaped groove structure arranged along the flow direction (the x-axis). The height and width of the V-shaped groove are denoted by *h* and *s*, respectively. Two rows of cubic columns are designed on the upper and lower walls near the inlet to generate rapidly developing turbulence. The length s′, width t, and height h′ of single cubic column are 0.5 mm.

### 2.3. Mesh Generation

**Figure 2:**
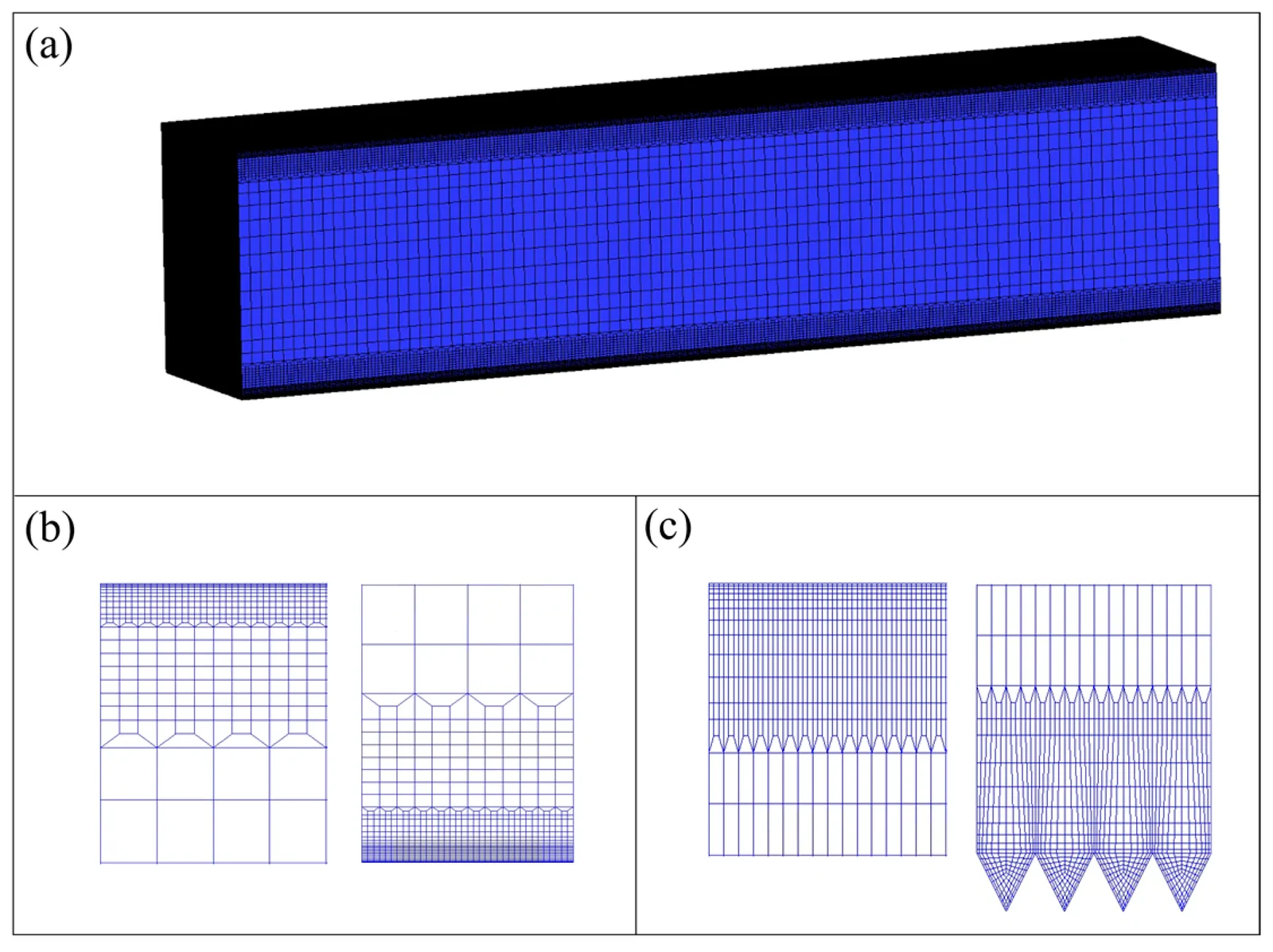
Mesh of the computational domain: (**a**) background mesh for the simulation; (**b**) the mesh near the smooth wall; (**c**) the mesh near the V-shaped groove wall.

The research on drag reduction of microstructural surfaces requires more attention to the flow field structure in the near-wall region. To minimize the influence of mesh quantity on the calculation results as much as possible, this paper employs a grid discretization scheme. The mesh in the near-wall region along the normal direction needs to be refined. The meshes away from the wall gradually become coarser. The meshes are uniformly distributed in the streamwise and spanwise directions. A Y-block division is adopted in the V-shaped groove. The meshes above the tip of the groove are identical to those of the smooth wall. Figure 2 illustrates the mesh details of the computational domain, which serves as the background mesh for the simulation.

The dimensionless mesh spacing $y^{+}$ in the *y*-direction can be calculated by the following formula.

(9) $$y^{+}=y\cdot \frac{u_{\tau }}{\nu }$$

(10) $$u_{\tau }=\sqrt{\frac{\tau_{w}}{\rho }}$$

(11) $$\tau_{w}=\frac{1}{2}C_{f}\rho U^{2}$$

(12) Cf= 2log10Rex−0.65−2.3

Here $\tau_{w}$ is the local wall shear stress. *C_f_* is the skin friction coefficient.

Table 2 presents the dimensionless mesh parameters near the wall region of the computational model. $x^{+}$ and $z^{+}$ are the dimensionless mesh spacings of the computational model in the *x* and *z* directions, respectively. ${y_{v}^{+}}_{\min}$ and ${y_{v}^{+}}_{\max}$ represent the minimum and maximum dimensionless mesh spacings in the *y*-direction inside the V-shaped groove wall, respectively. ${y^{+}}_{\min}$ denotes the minimum dimensionless mesh spacing in the *y*-direction for the smooth wall. These values can be predicted using Equation (9). *y^+^* < 1, *x^+^* < 40 and *z^+^* < 20 meet the size requirements for the first layer wall mesh of large eddy simulation (LES).

### 2.4. Grid Independence

At a frictional Reynolds number of 252, 591, and 695, the LES WALE subgrid model is used to verify the accuracy of the theoretical and simulated values of the C_f_. The theoretical value can be derived from Equation (13). As can be seen from Table 3, the relative error between the numerical simulation results and the theoretical values is small, which meets the requirements of calculation precision and accuracy. This demonstrates that the LES WALE subgrid model is suitable for simulating the turbulent boundary layer flow field over a flat plate.

(13) Cf=0.0742ReL5

(14) ReL =ρ U Lμ

${Re}_{L}$ is the global Reynolds number based on the characteristic length L.

### 2.5. Boundary Conditions and Numerical Simulation Methods

A quantitative analysis of drag reduction performance is achieved through high-precision numerical simulation methods. The inlet boundary condition is specified as a synthesized-eddy-based velocity. The outlet boundary condition is set as pressure. The boundary conditions on both sides are set to be cyclicAMI. The smooth wall, V-shaped groove wall, and the cubic columns are subject to no slip.

The standard *k-ω* model and the simpleFoam solver are employed for steady-state calculations. The kqRWallFunction wall function is used for the wall boundary. The turbulent dissipation rate ω is specified with the omegaWallFunction. The turbulent viscosity is set to nutUSpaldingWallFunction. The pressure boundary condition is set to zeroGradient. After the steady-state calculation converges, the mean velocity field, Reynolds-stress tensor field, and integral-length scale field on the vertical cross-section near the outlet are computed within the turbulence fields. The calculation results on this cross-section are taken as the initialization conditions for the velocity inlet boundary in the transient simulation. The pimpleFoam transient solver, the LES method, and the WALE subgrid model are employed in the transient calculations. The total turbulent kinetic energy (total TKE, $k_{\mathrm{total}}$) is calculated within the turbulence fields. The total TKE in the near-wall regions of the smooth and V-shaped groove wall on a certain cross-section show little difference, indicating that the inlet turbulent conditions for the smooth wall and grooved wall are identical. This cross-section is taken as the initial plane for analyzing the flow field drag characteristics. The results obtained from LES simulations are statistical values based on multiple calculation cycles. Therefore, this paper analyzes the results from five calculation cycles. The calculation formula for the time step *Δt* is as follows.

(15) $$\Delta t=c\;\frac{\Delta l}{U}$$

Courant number (*c*) is 0.8, and *Δl* represents the grid size.

### 2.6. Selection of the Initial Plane for Channel Flow

**Figure 3:**
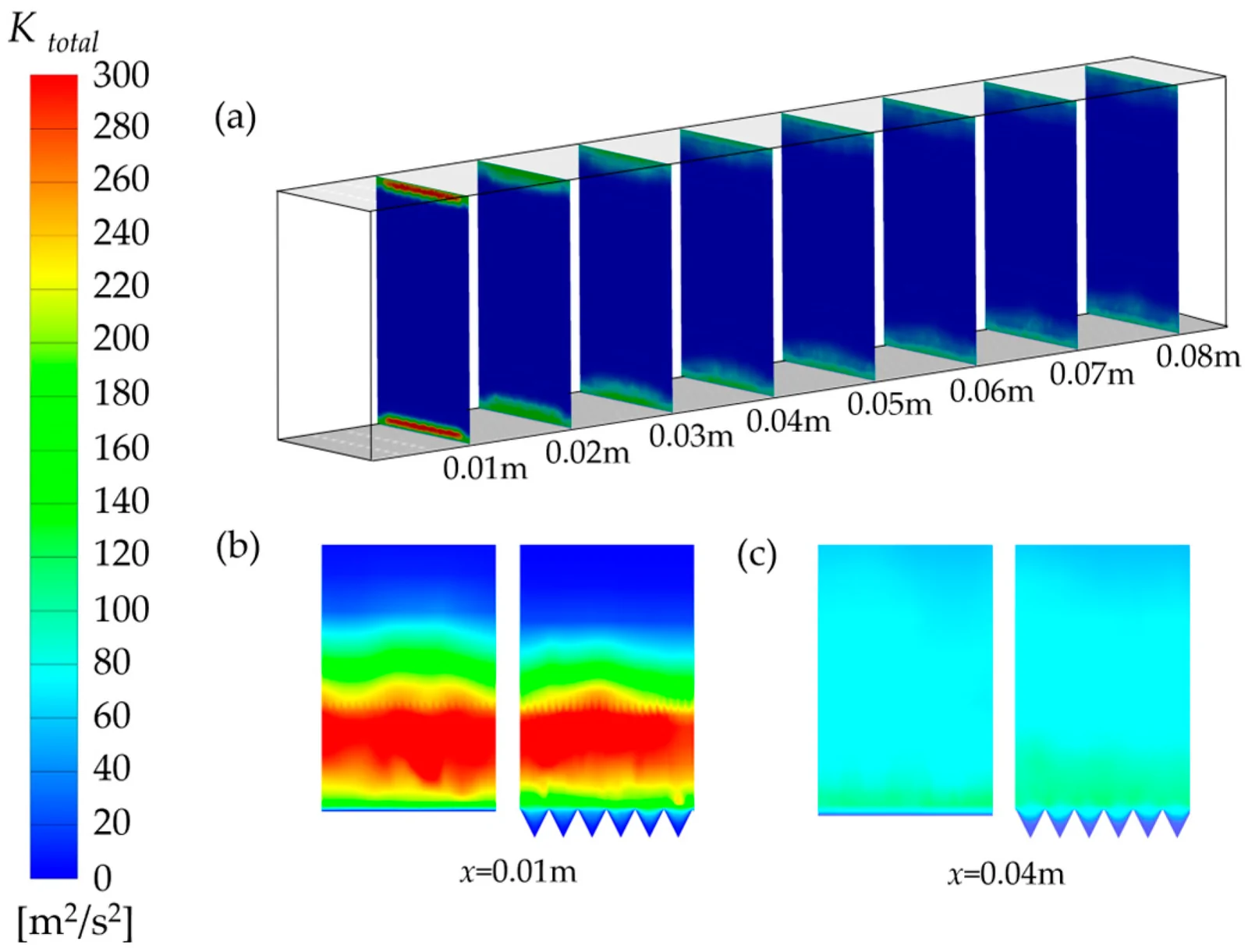
Total TKE contours of the computational domain: (**a**) Total TKE at cross-sections *x* = 0.01, 0.02, 0.03, 0.04, 0.05, 0.06, 0.07, and 0.08 m; (**b**,**c**) Total TKE near the wall at *x* = 0.01 m and 0.04 m; left: smooth wall, right: V-shaped groove wall.

To study fully developed turbulence, a plane at a certain distance from the inlet is intercepted as the initial plane, and the data on this plane is used as the initial value for analyzing the flow field. The greater the inlet velocity, the more unstable the flow field becomes in the same size of computational domain. Therefore, the cross-section of the flow field is obtained at a maximum frictional Reynolds number of 695. Planes at distances of *x* = 0.01 m, 0.02 m, 0.03 m, 0.04 m, 0.05 m, 0.06 m, 0.07 m, and 0.08 m from the inlet are intercepted along the streamwise direction of the computational domain. Figure 3 shows the distributions of total TKE on the V-shaped groove and smooth walls in theses eight sections. The formula for turbulent kinetic energy is as follows:

(16) $$k_{\mathrm{total}}=\frac{1}{2}\left(\bar{{\left(u’\right)}^{2}}+\bar{{\left(v’\right)}^{2}}+\bar{{\left(w’\right)}^{2}}\right)$$

u’, v’, w’ represent the components of the fluctuating velocity in the streamwise, normal, and spanwise directions, respectively. The component of the turbulent pulsation velocity is the difference between the instantaneous velocity and the mean velocity, and its formula is as follows:

(17) $$u’\;=\;u\;-\;\bar{u}$$

(18) $$v’=v-\bar{v}$$

(19) $$w’=w-\bar{w}$$

u,  v, and w represent the instantaneous velocities in the streamwise, normal, and spanwise directions, respectively. $\bar{u}$, $\;\bar{v}$, $\bar{\;w}$ represent the mean velocities respectively. The formulas for the mean values of fluctuating velocities $\bar{u’}$, $\;\bar{v’}$, $\;\bar{\;w’}$ are as follows:

(20) $$\bar{u’}\;=\;\frac{1}{T}\int_{0}^{T}\left(u\left(t\right)\;-\;\bar{u}\right)\mathrm{dt}$$

(21) $$\bar{v’}=\frac{1}{T}\int_{0}^{T}\left(v\left(t\right)-\bar{v}\right)\mathrm{dt}$$

(22) $$\bar{w’}=\frac{1}{T}\int_{0}^{T}\left(w\left(t\right)-\bar{w}\right)\mathrm{dt}$$

The formulas for the variances of fluctuating velocities $\bar{{\left(u’\right)}^{2}}$, $\;\bar{{\left(v’\right)}^{2}}$, $\bar{{\;\left(w’\right)}^{2}}$ are as follows:

(23) $$\bar{{\left(u’\right)}^{2}}\;=\;\frac{1}{T}\int_{0}^{T}{\left(u\left(t\right)\;-\;\bar{u}\right)}^{2}\mathrm{dt}$$

(24) $$\bar{{\left(v’\right)}^{2}}=\frac{1}{T}\int_{0}^{T}{\left(v\left(t\right)-\bar{v}\right)}^{2}\mathrm{dt}$$

(25) $$\bar{{\left(w’\right)}^{2}\;}=\frac{1}{T}\int_{0}^{T}{\left(w\left(t\right)-\bar{w}\right)}^{2}\mathrm{dt}$$

The total kinetic energy of turbulence ($k_{\mathrm{total}}$) can be derived from the sub-grid Reynolds stress tensor ($k_{\mathrm{sub-grid}}$) and the analytical flow Reynolds stress tensor ($k_{\mathrm{resolved}}$):

(26) $$k_{\mathrm{total}}\;={\;k}_{\mathrm{sub-grid}}\;+{\;k}_{\mathrm{resolved}}\;=\;\frac{1}{2}\mathrm{tr}\left(R\right)+\frac{1}{2}\mathrm{tr}\left(U\mathrm{Pr}i\mathrm{me}2\mathrm{Mean}\right)$$

Figure 3a shows that the distribution of the total TKE is basically consistent on the smooth and V-shaped groove wall across the eight sections. The total TKE increases from a small value to a certain level and then decreases. The cubic columns cause an increase of the total TKE near the inlet, such as at the cross-section of *x* = 0.01 m. The variation of the total TKE is relatively small in the cross-sections from *x* = 0.04 m to *x* = 0.08 m. Figure 3b shows that the total TKE first increases and then decreases in the two near-wall regions of the cross-section at *x* = 0.01 m. Figure 3c shows the variation of the total TKE on the smooth and V-shaped groove wall at the cross-section of *x* = 0.04 m, and its trend is consistent with that of the total TKE at the cross-section of *x* = 0.01 m. However, the range of variation in the values is relatively small.

**Figure 4:**
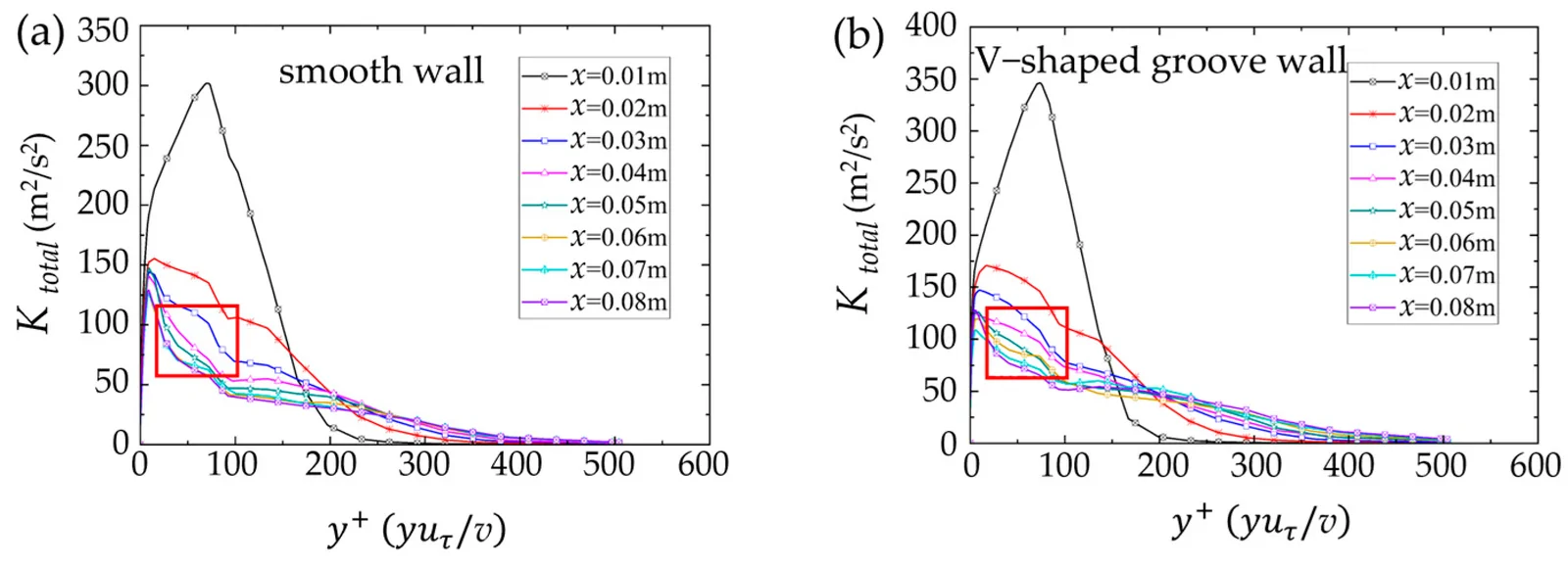
Total TKE on the normal vertical line at z = 0.004875 m for the eight planes: (**a**) smooth wall; (**b**) V-shaped groove wall. The red-boxed region illustrates the variation of the total TKE for the smooth wall and V-shaped groove wall when *y^+^* ranges from 10 to 100.

Figure 4a shows that the total TKE increases sharply to a peak value and then decreases on the normal vertical line at z = 0.004875 m of the smooth wall. At the cross-section of *x* = 0.01 m, the trend of the total TKE is relatively large. The position where the peak value occurs is the farthest from the wall. The variations of total TKE at the cross-sections of *x* = 0.04 m, 0.05 m, 0.06 m, 0.07 m, and 0.08 m are consistent. In Table 4, the total TKE is 305 m^2^/s^2^ at the cross-section of *x* = 0.01 m, where the dimensionless distance *y^+^* from the smooth wall is 72.15. As the fluid develops along the inflow direction, the peak values of total TKE gradually decrease. The peak values of total TKE on the normal vertical lines at the cross-sections of *x* = 0.04 m, 0.05 m, 0.06 m, 0.07 m, and 0.08 m are not greatly different. Figure 4b shows the distributions of the total TKE near the V-shaped groove wall, and its variations are consistent with those of the smooth wall. The total TKE is 350 m^2^/s^2^ at the cross-section of *x* = 0.01 m, where the dimensionless distance *y^+^* from the V-shaped groove wall is 73.23. The positions of the peak values of total TKE on the normal vertical lines at the cross-sections of *x* = 0.04 m, 0.05 m, 0.06 m, 0.07 m, and 0.08 m for the V-shaped groove wall are close to those of the smooth wall. It can be seen from Table 4 that when the dimensionless distance *y^+^* from the wall is approximately 9, the peak values of total TKE between the smooth wall and the V-shaped groove wall are not significantly different. Therefore, the law of drag reduction and its mechanism on the V-shaped groove wall are analyzed in the cross-sectional region from *x* = 0.04 m to *x* = 0.08 m.

## 3. Analysis of the Law of Drag Reduction in the Grooved Flat Channel

Table 5 shows the drag reduction rate of V-shaped groove wall under different micro-structural sizes. The V-shaped groove structure achieves drag reduction with dimensionless height and width (*h^+^* and *s^+^*) both ranging between 8.50 and 29.75. At a velocity of 80.7 m/s, *h^+^* = *s^+^* = 25.29, the V-shaped groove wall achieves the maximum drag reduction rate (*DR*). $D_{s}$ represents the drag force of the smooth flat plate, while $D_{r}$ denotes the drag force of the grooved plate. Eight repeated numerical calculations are performed for Group 15. The drag reduction rates from the calculation results show little deviation for that of this group, with all values around 0.7.

(27) $$D_{s}=\mu \int_{A_{s}}\frac{\partial u}{\partial y}dA_{s}$$

(28) $$D_{r}=\mu \int_{A_{r}}\frac{\partial u}{\partial y}dA_{r}$$

(29) $$\mathrm{DR}(\%)=\frac{{\bar{D}}_{s}-{\bar{D}}_{r}}{{\bar{D}}_{s}}\;\times \;100$$

Here *A_s_* and *A_r_* represent the areas of the smooth wall and the groove wall, respectively. ${\bar{D}}_{s}$ and ${\bar{D}}_{r}$ represent the time-averaged resistance of the smooth and the groove wall, respectively. The averaging duration used for statistical analysis is 0.2 s.

The relationship formula between the height *h*, width *s*, inflow velocity *U* and the drag reduction rate is as follows:

*DR*(*%*) = −18.402 + 63.438*h* + 41.497*s* + 0.995*U* − 2.138*hU* − 2.298*sU* − 0.004*U*^2^ (30)

The coefficient of determination *R*^2^ = 0.9753, indicating that the groove height *h*, groove width *s*, inlet velocity *U*, and their interaction terms dominate the variation of drag reduction rate. Adjusted coefficient of determination $R_{\mathrm{adj}}^{2}$ = 0.9632, indicating no invalid redundant variables in the regression formula. The ANOVA results show that the overall model F = 85.74 and *p* < 0.0001, proving the regression model is highly statistically significant.

### 3.1. Interaction Effects on Drag Reduction Rate When One of the Three Variables Is Fixed at Its Medium Value

**Figure 5:**
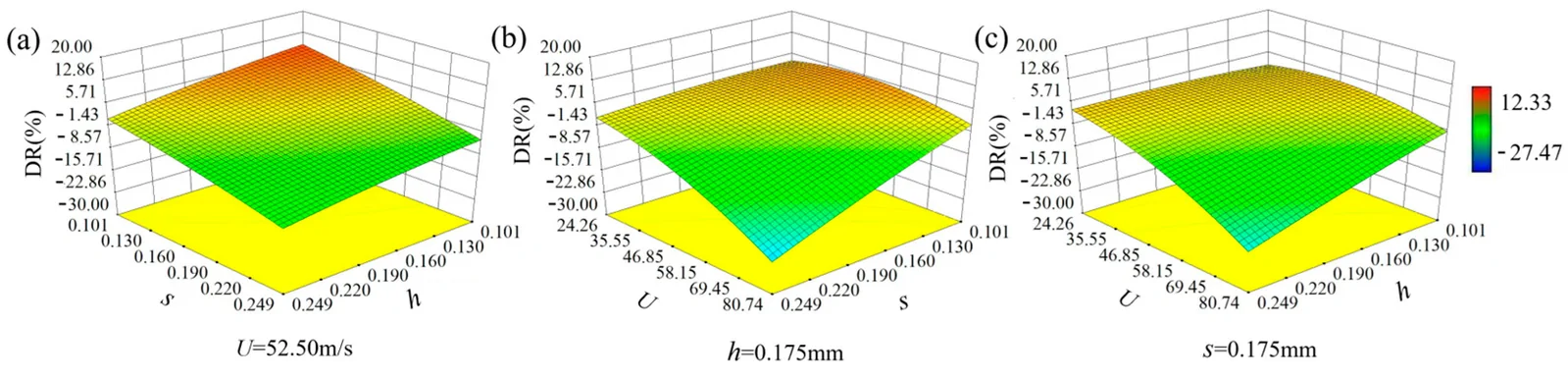
Interaction effects on drag reduction rate when one of the three variables (*h*, *s*, *U*) is fixed at its medium value. (**a**) The influence of the height *h* and width *s* of V-shaped grooves on the drag reduction rate when the inflow velocity *U* is 52.50 m/s. (**b**) The influence of the inflow velocity *U* and width *s* of V-shaped grooves on the drag reduction rate when the height *h* is 0.175 mm. (**c**) The influence of the inflow velocity *U* and the height *h* of V-shaped grooves on the drag reduction rate when the width *s* is 0.175 mm.

The analysis of response surface is conducted to investigate the relationship between *h*, *s*, *U*, and drag characteristics. Figure 5 shows the interaction effects on drag reduction rate when one of the three variables (*h*, *s*, *U*) is fixed at its medium value. When the velocity is 52.50 m/s, and the groove height and groove width are both in the ranges of 0.101–0.160 mm, the drag reduction rate of the V-shaped groove wall is relatively high. When the groove height is 0.175 mm, the groove width ranges from 0.101 mm to 0.160 mm, and the inflow velocity is between 35.55 m/s and 58.15 m/s, the V-shaped groove wall achieves a favorable drag reduction effect. When the groove width is 0.175 mm, the groove height ranges from 0.101 mm to 0.220 mm, and the inflow velocity is between 24.26 m/s and 46.85 m/s, the V-shaped groove wall exhibits a significant drag reduction effect.

### 3.2. Interaction Between Groove Height and Width on Drag Reduction Rate at a Constant Velocity

**Figure 6:**
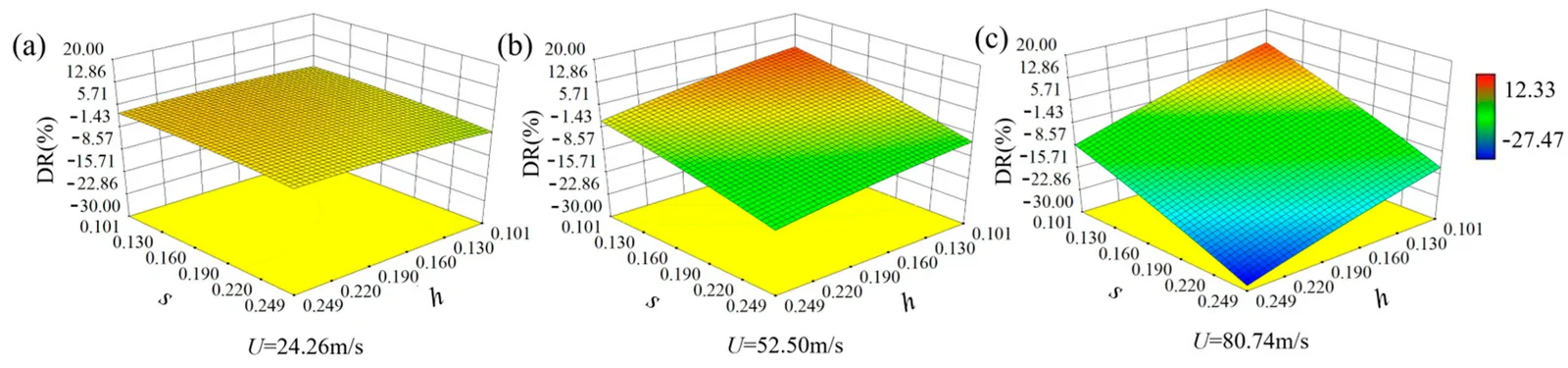
Interaction between groove height and width on drag reduction rate at *U* = 24.26, 52.50, and 80.74 m/s. (**a**) The influence of the height *h* and width *s* of V-shaped grooves on the drag reduction rate under inflow velocity of 24.26 m/s. (**b**) The influence of the height *h* and width *s* of V-shaped grooves on the drag reduction rate under inflow velocity of 52.50 m/s. (**c**) The influence of the height *h* and width *s* of V-shaped grooves on the drag reduction rate under inflow velocity of 80.74 m/s.

Figure 6 shows the interaction between groove height and width on drag reduction rate at *U* = 24.26, 52.50, and 80.74 m/s. At a velocity of 24.26 m/s, groove height is positively correlated with the drag reduction rate, while groove width is negatively correlated with it. The correlation between two variables and the drag reduction rate is relatively weak. The drag reduction rate reaches its optimal value when the groove height is 0.249 mm and the groove width is 0.101 mm. At velocities of 52.50 m/s and 80.74 m/s, both groove height and groove width exhibit a negative correlation with drag reduction rate. At a velocity of 80.74 m/s, the negative correlation is significant, and there is even an increase in drag. At these two velocities, the V-shaped groove wall shows a significant drag reduction effect when the groove height and width are in the ranges of 0.101–0.130 mm. The drag-reduction effect is optimal when both the groove height and width are set to their minimum values of 0.101 mm.

### 3.3. Interaction Between Groove Height and Velocity on Drag Reduction Rate at a Groove Width

**Figure 7:**
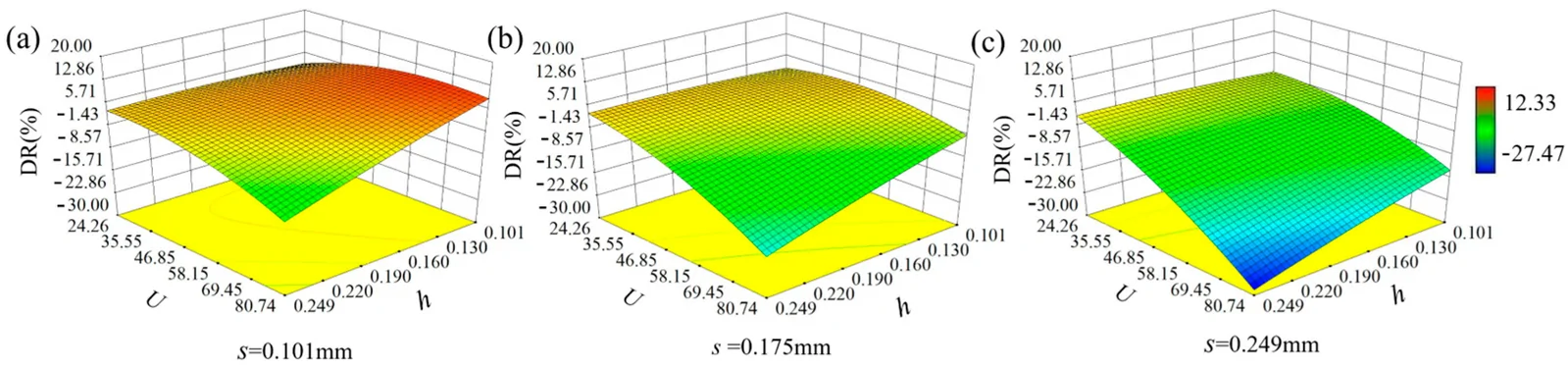
Interaction between groove height and velocity on drag reduction rate for *s* = 0.101, 0.175, and 0.249 mm. (**a**) The influence of the inflow velocity *U* and height *h* of V-shaped grooves on the drag reduction rate under width *s* of 0.101 mm. (**b**) The influence of the inflow velocity *U* and height *h* of V-shaped grooves on the drag reduction rate under width *s* of 0.175 mm. (**c**) The influence of the inflow velocity *U* and height *h* of V-shaped grooves on the drag reduction rate under width *s* of 0.249 mm.

Figure 7 shows the interaction between groove height and velocity on drag reduction rate for groove width *s* = 0.101, 0.175, and 0.249 mm. When the groove width is 0.101 mm, the groove height ranges from 0.101 mm to 0.130 mm, and the velocity ranges from 58.15 m/s to 80.74 m/s, the groove wall exhibits a high drag reduction effect. When the groove width is 0.175 mm, the groove height ranges from 0.101 mm to 0.190 mm, and the inflow velocity ranges from 35.55 m/s to 46.85 m/s, the drag reduction rate of the V-shaped groove wall reaches its maximum value. When the groove width is 0.249 mm, the groove height is over the entire range, and the velocity is 24.26–35.55 m/s, the drag reduction rate of the V-shaped groove wall is relatively high.

### 3.4. Interaction Between Groove Width and Velocity on Drag Reduction Rate at a Groove Height

**Figure 8:**
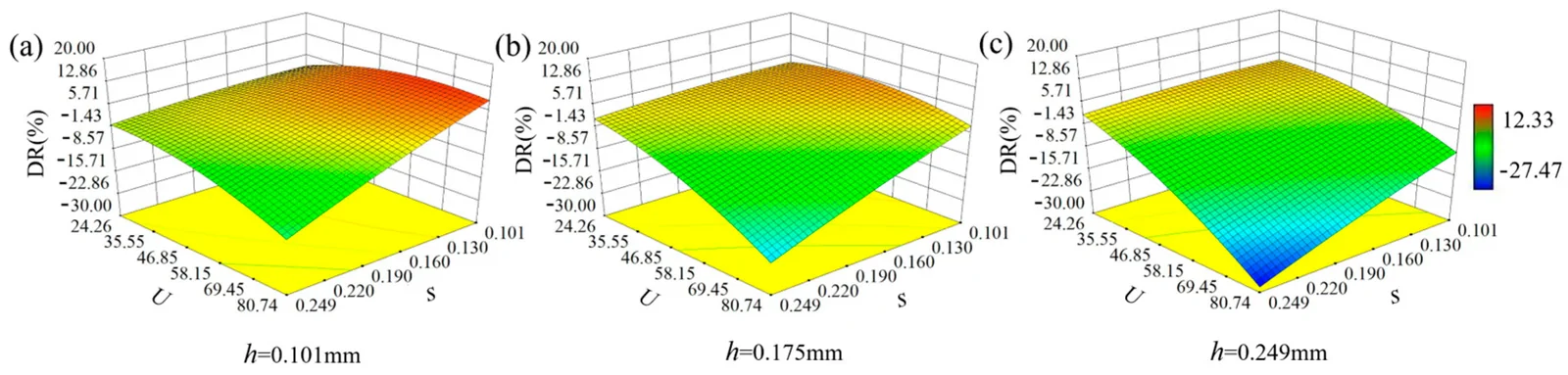
Interaction between groove width and velocity on drag reduction rate for groove height *h* = 0.101, 0.175, and 0.249 mm. (**a**) The influence of the inflow velocity *U* and width *s* of V-shaped grooves on the drag reduction rate under height *h* of 0.101 mm. (**b**) The influence of the inflow velocity *U* and width *s* of V-shaped grooves on the drag reduction rate under height *h* of 0.175 mm. (**c**) The influence of the inflow velocity *U* and width *s* of V-shaped grooves on the drag reduction rate under height *h* of 0.249 mm.

Figure 8 shows the interaction between groove width and velocity on drag reduction rate for groove height *h* = 0.101, 0.175, and 0.249 mm. When the groove height is 0.101 mm, the groove width is 0.101–0.130 mm, and the velocity is 58.15–80.74 m/s, the drag reduction effect on the groove wall is significant. When the groove height is 0.175 mm, the groove width ranges from 0.101 mm to 0.160 mm, and the velocity ranges from 35.55 m/s to 58.15 m/s, the groove wall exhibits a good drag-reduction effect. When the groove height is 0.249 mm, the groove width is 0.101–0.160 mm, and the velocity is 24.26–35.55 m/s, the drag reduction rate of the V-shaped groove wall is relatively large.

Analysis of the above results reveals the laws of drag reduction in the grooves. At different velocities, appropriate microgroove dimensions must be designed to achieve drag reduction. For example, the groove height and width are relatively large under low-speed conditions, while they decrease under medium-to-high-speed conditions. In the range of velocity (5–100 m/s), the higher the incoming flow velocity, the smaller the groove dimensions.

### 3.5. Comparison with Previous Research Results

Table 6 shows a comparison between the results of this study and previous research. For conventional continuous two-dimensional V-shaped riblets mounted on the flat plate of an oil channel test facility, the optimal dimensionless riblet spacing *s^+^* = 17, optimal dimensionless riblet height *h^+^* = 8.4, with a maximum drag reduction rate of 9.01% [10]. Symmetrical longitudinal V-shaped riblets with a vertex angle of 40° are mounted on the flat floor of the low-speed wind tunnel. A maximum drag reduction rate of 3% is achieved at the dimensionless riblet height *h^+^* = 13 and dimensionless spacing *s^+^* = 20 [13]. For standard 60° triangular riblets, the optimal dimensionless spacing *s^+^* = 17.3 and dimensionless height *h^+^* = 8.6 are obtained, with a maximum skin-friction drag reduction rate of 8.7% [18]. A symmetrical V-shaped longitudinal grooved rib structure was arranged on the flat wall of a low-speed turbulent wind tunnel. With the optimized dimensionless groove spacing *s^+^* = 12 and groove height *h^+^* = 12, the maximum drag reduction rate is 8% [25]. 60° triangular streamwise riblets are arranged on the flat wall of turbulent channel and wind tunnel test facilities. The optimal dimensionless riblet spacing *s^+^* is15 and the corresponding optimal dimensionless riblet height *h^+^* = 7.5, achieving a maximum drag reduction rate of 8% [26]. In this work, biomimetic equal-height symmetrical V-shaped grooves mimicking shortfin mako shark denticles are arranged on the bottom flat wall of a turbulent channel. The optimal dimensionless riblet spacing *s^+^* = 25.29 and dimensionless height *h^+^* = 25.29 are obtained, achieving a maximum turbulent skin-friction drag reduction rate of 12.33%. A quantitative comparison reveals that the biomimetic V-groove geometry proposed in this work achieves a markedly higher maximum drag reduction rate compared with conventional riblet configurations reported in prior literature.

## 4. Analysis of the Mechanism of Drag Reduction in Grooved Flat Channel

### 4.1. Vorticity Distribution and Velocity Streamline Plots

**Figure 9:**
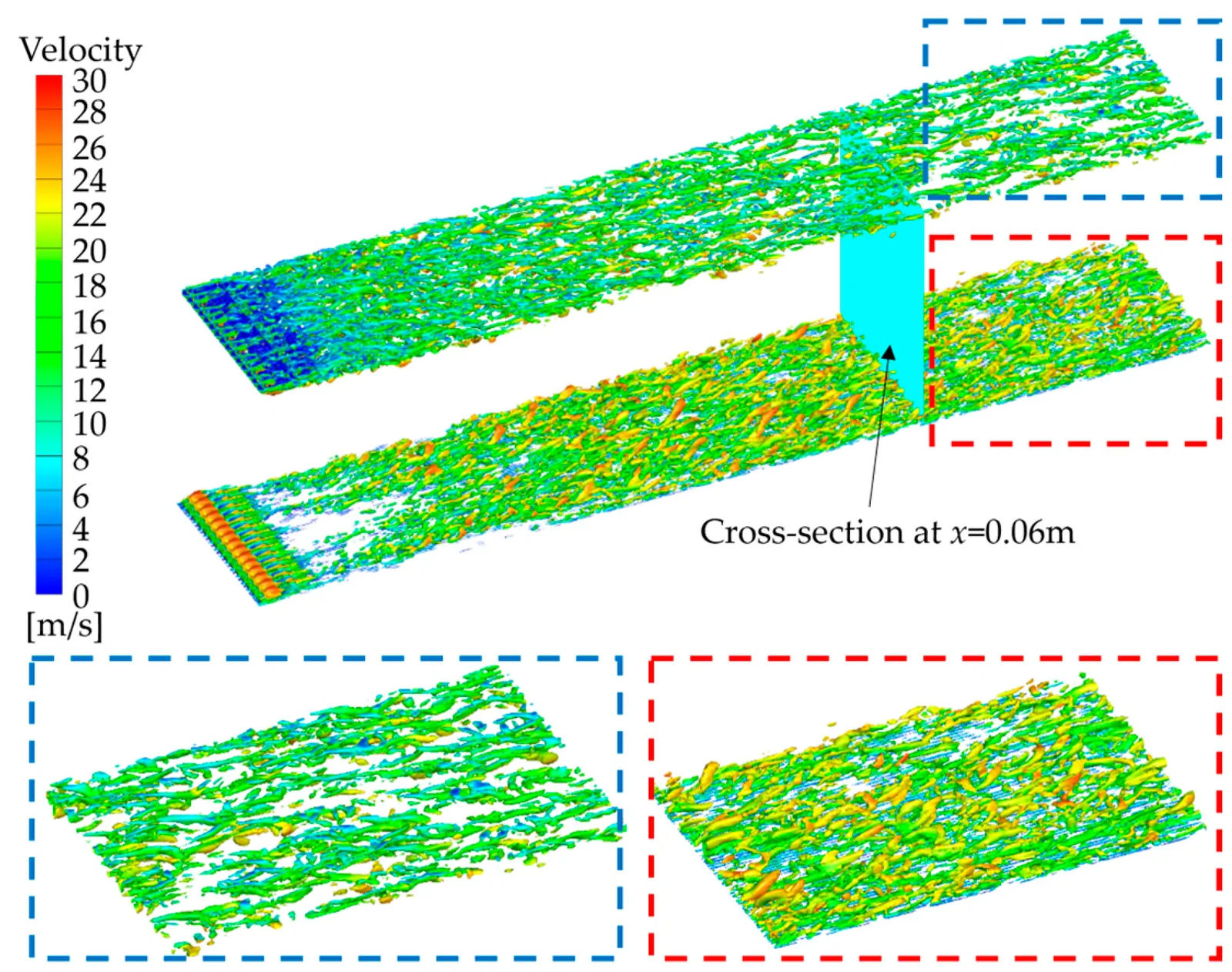
Vorticity variations (Q-criterion) of the smooth wall and V-shaped groove wall.

Figure 9 illustrates the vorticity variations (Q-criterion) of the smooth wall and V-shaped groove wall under an incoming flow velocity of 24.3 m/s based on case 3 in Table 5. The color of the vortex is represented by velocity. Large-scale and high-intensity streamwise vortices exist in the near wall region of the smooth wall. The vortices sweep downward and strike the wall, resulting in intense momentum exchange and high shear stress on the wall. This is the primary source of high drag in turbulence. The V-shaped grooves break high-intensity vortices into small, low-velocity, weak vortices. The size of the vortex and its peak flow velocity decreased significantly. The intensity of high-momentum events such as ejection and sweep is greatly attenuated. The V-shaped grooves act as small fences, suppressing the movement of transverse turbulent vortices near the wall, which is consistent with Choi’s findings [13].

**Figure 10:**
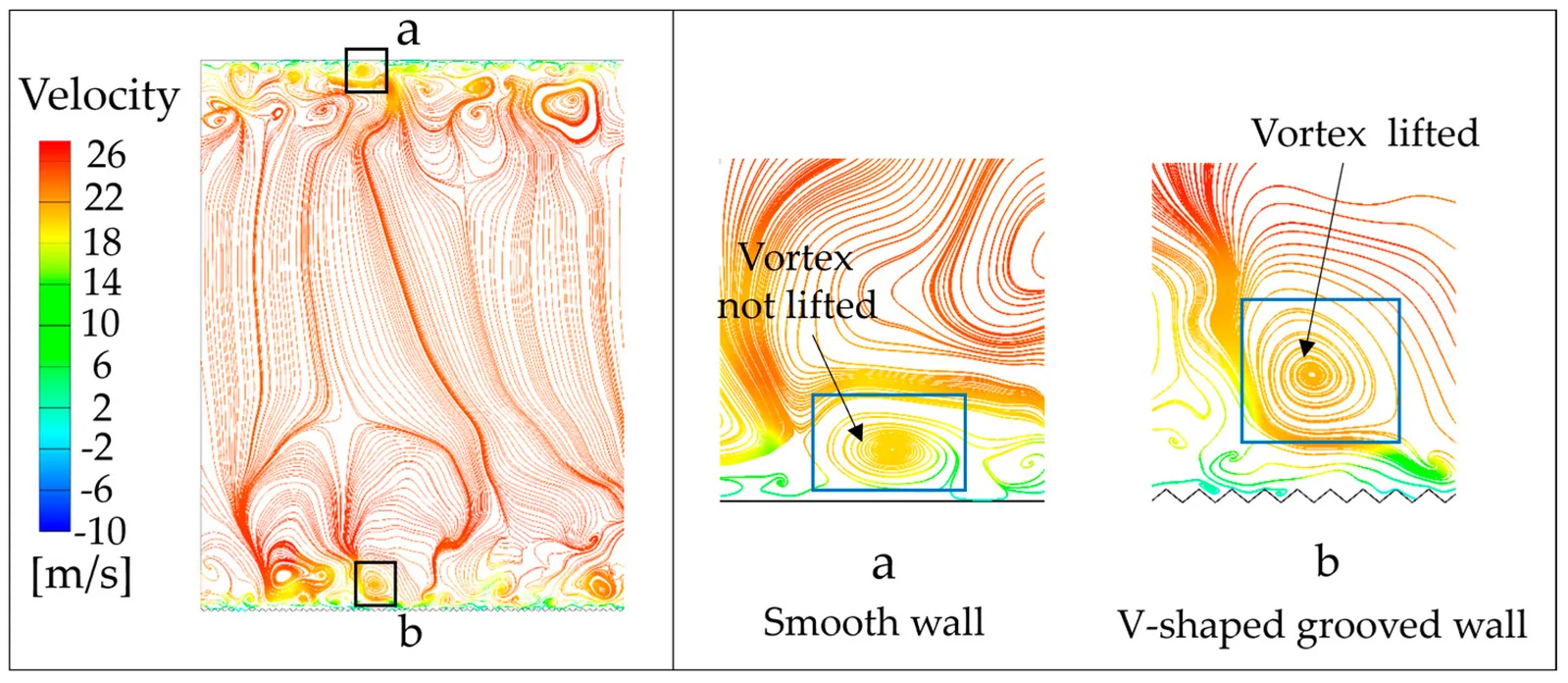
The velocity streamline distribution on the cross-section at *x* = 0.06 m.

Figure 10 shows the velocity streamline distribution on the cross-section at *x* = 0.06 m presented in Figure 9. The core of coherent vortex on the smooth wall is located closer to the wall, and the vortex is not lifted. The vortex rotates along the axis in the direction of the flow velocity, generating jet and sweeping motions, mixing between high-speed and low-speed fluids near the wall. Consequently, this motion creates regions of high shear stress. The vortices on the V-shaped groove wall are lifted, and the low-speed fluid thickens the viscous boundary layer. This indicates that an appropriate groove size suppresses momentum transport of turbulent pulsations, reduces the average shear stress at the wall, and ultimately achieves a drag reduction in the turbulent boundary layer.

### 4.2. The Average Velocity Distribution in the Boundary Layer of a Grooved Flat Channel

**Figure 11:**
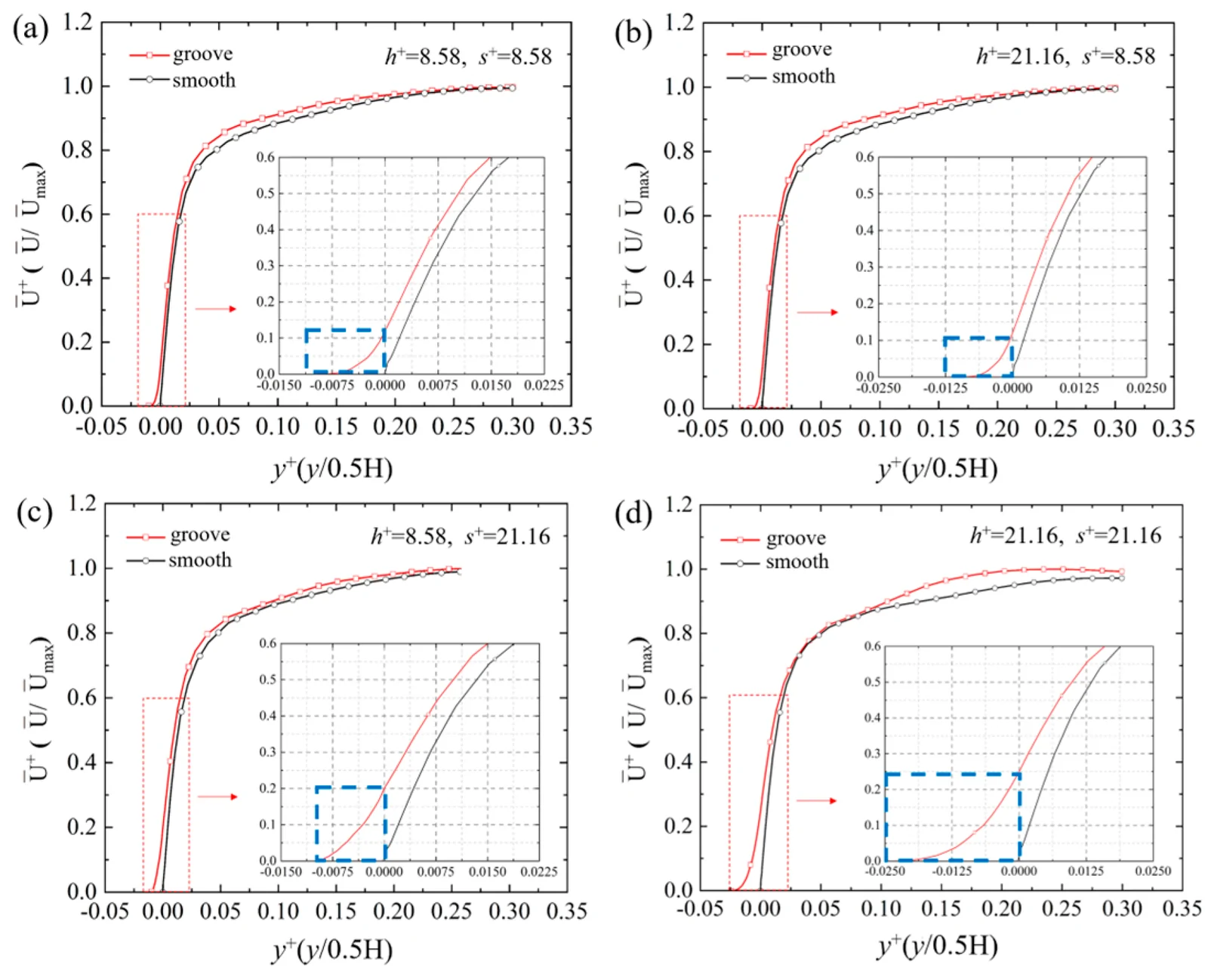
Dimensionless average velocity distribution along the normal direction of the groove and smooth wall at a velocity of 24.3 m/s: (**a**) Case 3, *h^+^* = *s^+^* = 8.58; (**b**) Case 2, *h^+^* = 21.16, *s^+^* = 8.58; (**c**) Case 7, *h^+^* = 8.58, *s^+^* = 21.16; (**d**) Case 4, *h^+^* = *s^+^* = 21.16, *y^+^* = *y*/0.5H, ${\bar{U}}^{+}\;$=$\bar{\;U}$/${\bar{U}}_{max}$. The blue diagram represents the area inside the groove.

Based on the cases 2, 3, 4, and 7 in Table 5, a comparative analysis is conducted to examine the effect of V-shaped groove structures of different sizes on the velocity in the boundary layer at a velocity of 24.3 m/s. As shown in Figure 11, the trends in the dimensionless average flow velocity curves for the V-shaped groove and the smooth wall are essentially consistent. The average flow velocity in the flow field near the wall increases rapidly. When the dimensionless average flow velocity ranges from 0 to 0.8, the velocity gradient (Δ${\bar{U}}^{+}$/Δ *y^+^*) remains high. The average flow velocity away from the wall gradually stabilizes. In Figure 11a, the average flow velocity inside the groove is relatively low and varies slowly. However, the flow velocity increases rapidly upon reaching the groove tip. The average flow velocity on the smooth wall follows the same trend as the velocity at the tip of the groove. In Figure 11b–d, the variations in the average flow velocity of the groove and smooth wall are identical to those in Figure 11a. The velocity in the groove increases slowly, while the flow velocity above the tip of the groove increases sharply. The average velocity gradient near the wall on the groove wall is smaller than that on a smooth wall. This indicates the presence of a low-velocity flow zone in the groove. The micro-groove alters the turbulent structure of the flow field on the wall.

### 4.3. Pulsating Velocity

According to case 1 in Table 5, the groove structure has the highest drag reduction rate on the wall, approximately 12.33%. This computational model is selected to analyze the pulsating velocity in the flow field. The formation and development of turbulent vortices primarily occur in the viscous near-wall region (0 < *y^+^* < 50). The viscous near-wall region is closely related to turbulent mechanisms. Therefore, this study investigates the turbulent flow characteristics in the viscous near-wall region.

**Figure 12:**
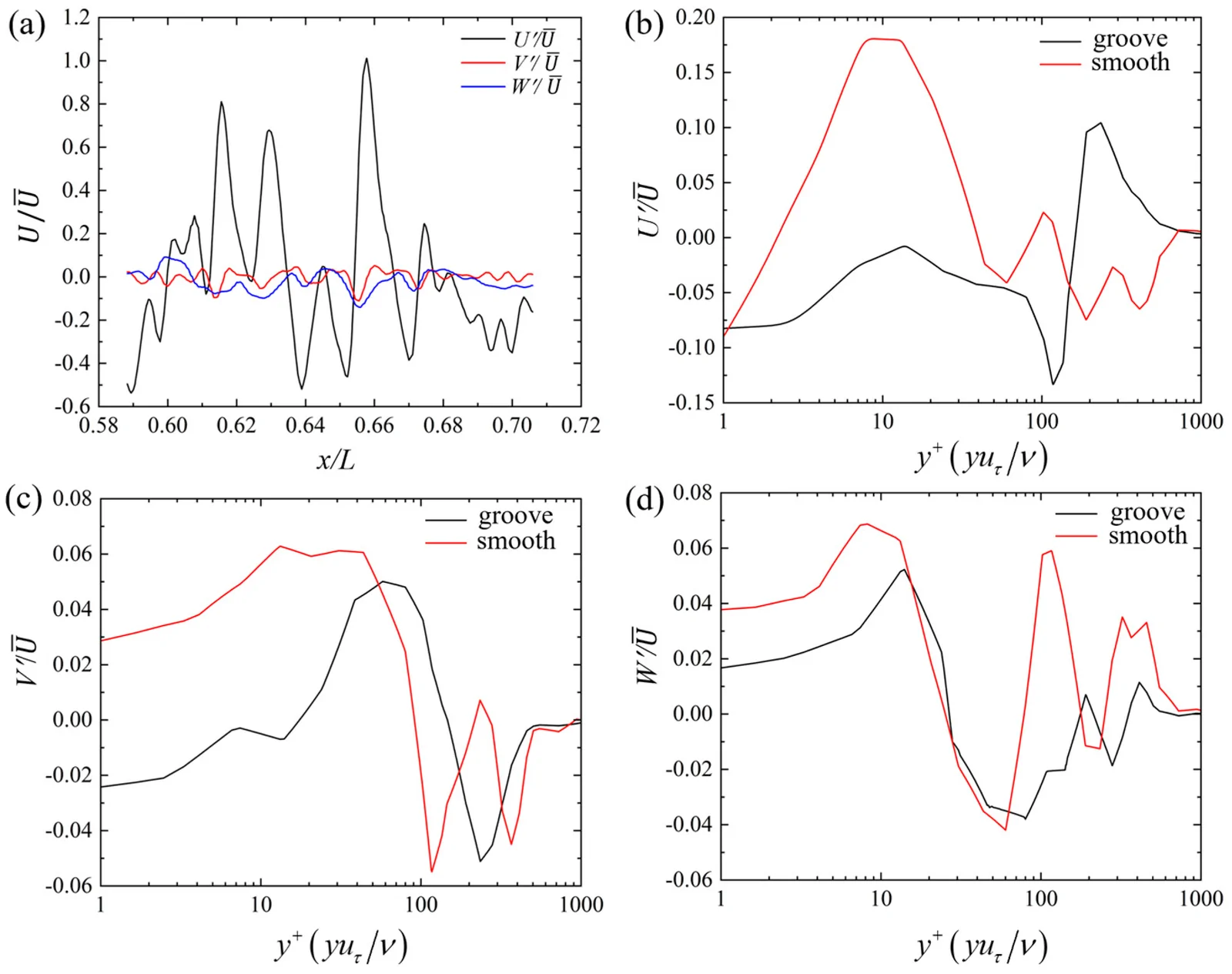
Pulsating velocity distributions: (**a**) variation of pulsating velocity along the streamwise direction at the center of the groove; (**b**) streamwise component on the smooth wall and above groove tip; (**c**) normal component on the smooth wall and above groove tip; (**d**) spanwise component on the smooth wall and above groove tip.

Figure 12a shows the pulsating velocity along the streamwise direction at the center of the groove. The pulsating velocities in all three directions fluctuate around 0. The normal pulsating velocity (*V’*/$\bar{U}$) at the center of the channel is the smallest. The average of the absolute value of *V’*/$\bar{U}$ is 0.0234. The pulsating velocity in the spanwise direction (*W’*/$\bar{U}$) is slightly larger. The average of the absolute value of *W’*/$\bar{U}$ is 0.0428. The pulsating velocity in the streamwise direction (*U’*/$\bar{U}$) is the largest, and the average of the absolute value of *U’*/$\bar{U}$ is 0.2652. This indicates that the streamwise pulsating velocity is the largest among the three components and plays a dominant role.

Figure 12b shows the distribution of pulsating velocity in the streamwise direction on the smooth wall and the upper part of the groove tip. In the viscous sublayer (0 < *y^+^* < 5) and the buffer layer (5 < *y^+^* < 30), the absolute value of the pulsating velocity in the streamwise direction on the V-shaped groove wall is smaller than that on the smooth wall. The average value decreases from 0.1406 to 0.0347, a reduction of 0.1059. The peak in the absolute value of the flow pulsating velocity occurs at *y^+^* = 17, and the V-shaped groove structure reduces the *U’/*$\bar{U}$ from 0.1804 to nearly zero. In the logarithmic region (30 < *y^+^* < 50), the average value of the flow-direction pulsating velocity on the smooth wall is 0.0206, while that for the V-shaped groove wall is 0.0544, representing an increase of 0.0338. The increase in the absolute value of the flow-direction pulsating velocity in the logarithmic layer is much smaller than the decrease observed in the viscous sublayer and buffer layer. This indicates that the V-shaped groove structure significantly suppresses the flow-directional pulsating velocity in the viscous sublayer and buffer layer.

Figure 12c shows the distribution of pulsating velocity in the normal direction on the smooth wall and the upper part of the groove tip. In the viscous near-wall region (0 < *y^+^* < 50), the absolute value of the pulsating velocity in the normal direction on the V-shaped groove wall is smaller than that on the smooth wall. The average value decreases from 0.0561 to 0.0246, a reduction of 0.0315. The peak of the absolute value on the smooth wall occurs at *y^+^*= 13, while the peak value on the V-shaped groove wall occurs at *y^+^*= 87, indicating that the position of peak value shifts upward along the wall. This suggests that the V-shaped groove structure reduces fluctuations in the normal pulsating velocity within the viscous near-wall region, thereby suppressing turbulence. Figure 12d shows the distribution of pulsating velocity in the spanwise direction on the smooth wall and the upper part of the groove tip. When 0 < *y^+^* < 15 and 27 < *y^+^* < 50, the absolute value of the pulsating velocity in the spanwise direction is smaller on smooth wall than on V-shaped groove wall. The absolute value decreased from 0.0445 to 0.0298, a reduction of 0.0147. This indicates that the V-shaped groove structure can suppress the spanwise distribution of turbulent vortices.

The V-shaped groove structure restricts the development of the spanwise and normal pulsating velocity inside the groove. The pulsating velocity along the streamwise direction dominates in the flow field. The V-shaped groove structure reduces the pulsating velocity in all three directions, which is consistent with the results of DNS numerical simulations by Le et al. [27]. The V-shaped groove structure has the strongest inhibitory effect on the streamwise fluctuating velocity, a weaker inhibitory effect on the normal fluctuating velocity, and the weakest inhibitory effect on the spanwise fluctuating velocity. The peak value of pulsating velocities in all three directions on the V-shaped groove wall decrease and move away from the wall. This indicates that the V-shaped groove structure can reduce turbulence flow near the wall and increase the thickness of boundary layer. Chu and Karniadakis [28] also confirmed similar findings.

### 4.4. Reynolds Stress

The Reynolds stress in the flow field is analyzed based on case 1 in Table 5. Reynolds stress, also known as turbulent stress, is an additional stress caused by turbulent pulsations in momentum exchange. The dimensionless Reynolds stress ($\langle u_{i}^{’}u_{j}^{’}\rangle =\bar{u_{i}^{’}u_{j}^{’}}/U^{2}$) is defined as the ratio of the time average of the velocity fluctuations to the square of the incoming velocity.

**Figure 13:**
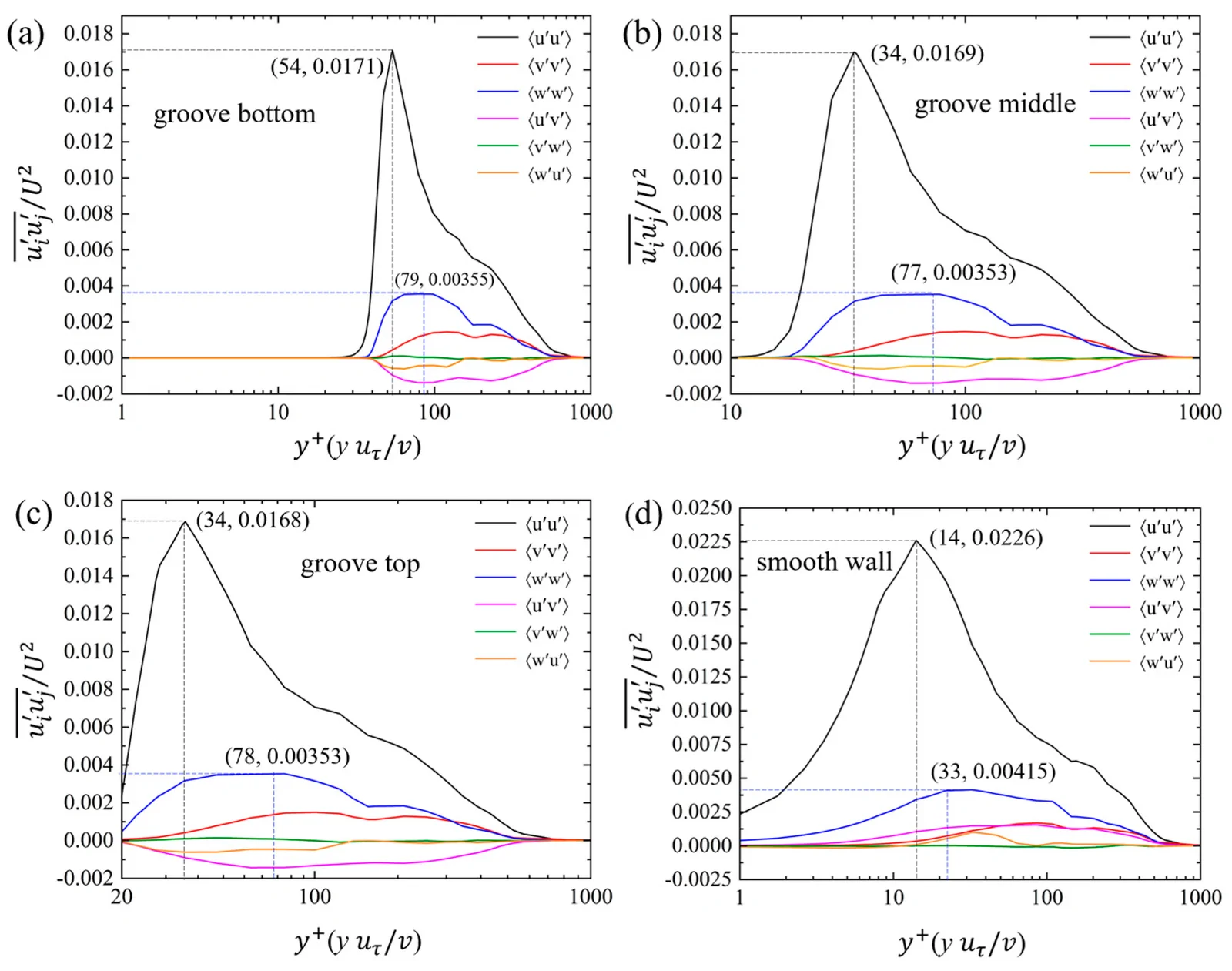
Distribution of Reynolds stress on the V-shaped groove and smooth wall: (**a**) groove bottom; (**b**) groove middle; (**c**) groove top; (**d**) smooth wall.

At the cross-section *x* = 0.06 m, the Reynolds stress on the V-shaped groove wall is obtained along three perpendicular lines at *z* = 4.798 mm (groove bottom), 4.823 mm (groove middle), and 4.848 mm (groove top). The Reynolds stresses along the perpendicular line corresponding to the top of the groove is extracted on the smooth wall. Figure 13 shows the distribution of Reynolds stress on the V-shaped groove and smooth wall. For both the V-shaped groove and smooth wall, the Reynolds normal stress ($\left\langle u^{’}u^{’}\right\rangle$) in the streamwise direction accounts for a large portion of the total stress.

For both the V-shaped groove and smooth wall, the Reynolds normal stress in the streamwise direction ($\left\langle u^{’}u^{’}\right\rangle$) is greatest, followed by the Reynolds normal stress in the spanwise direction ($\left\langle w^{’}w^{’}\right\rangle$). The normal Reynolds stress ($\left\langle v^{’}v^{’}\right\rangle$) is comparable to Reynolds shear stress in the streamwise direction ($\left\langle u^{’}v^{’}\right\rangle$). The effects of the other stress components are relatively small. In Figure 13a–c, the peak value of $\left\langle u^{’}u^{’}\right\rangle$ at the bottom, middle, and top of the groove are 0.0171, 0.0169, and 0.0168, respectively. The peak value of $\left\langle u^{’}u^{’}\right\rangle$ on the smooth wall is 0.0226 in Figure 13d. The peak value of $\left\langle u^{’}u^{’}\right\rangle$ at the bottom, middle, and top of the groove, are approximately 24.3%, 25.2%, and 25.7%, lower than those on a smooth wall. The grooved structure effectively reduces $\left\langle u^{’}u^{’}\right\rangle$ in the viscous near-wall region, and the turbulent pulsations in the boundary layer near the wall of the groove are suppressed. The peak value of $\left\langle u^{’}u^{’}\right\rangle$ at the bottom (*y^+^* = 54), middle (*y^+^* = 34), and top (*y^+^* = 34) of the groove occur in the logarithmic layer. However, the peak value of $\left\langle u^{’}u^{’}\right\rangle$ for the smooth wall (*y^+^* = 14) occurs in the buffer layer. The grooved structure weakens the energy exchange in turbulent flow between the viscous sublayer and the buffer layer. The peak value of $\left\langle w^{’}w^{’}\right\rangle$ for the bottom (*y^+^* = 79), middle (*y^+^* = 77), and top (*y^+^* = 78) of the grooved wall are 0.00355, 0.00353, and 0.00353, respectively. Compared to the peak value of $\left\langle w^{’}w^{’}\right\rangle$ (0.00415) for a smooth wall (*y^+^* = 33), the peak values of the grooved wall are reduced by 14.5%, 14.9%, and 14.9%, respectively. Turbulent vortices in the spanwise direction are hindered, meaning that longitudinal grooves can restrict the development of vortices in the spanwise direction. This is consistent with the description in the “second vortex group” theory [29].

Among the Reynolds stress components, the $\left\langle u^{’}u^{’}\right\rangle$ of flow field accounts for the major portion. Compared to a smooth wall, the V-shaped groove structure effectively reduces both $\left\langle u^{’}u^{’}\right\rangle$ and $\left\langle w^{’}w^{’}\right\rangle$ in the viscous near-wall region. The grooved structure effectively suppresses turbulent fluctuations in the flow field.

## 5. Conclusions

In this study, the turbulent drag reduction characteristics of V-shaped groove surfaces inspired by shark skin microstructures are systematically investigated using DOE method combined with high-precision numerical simulations. The effects of groove height (*h^+^*), groove width (*s^+^*), and inflow velocity (*U*) on drag reduction rate are quantified, and the flow mechanisms are elucidated through analyses of vorticity distribution, vortex core position boundary layer velocity distributions, pulsating velocity fields, and Reynolds stress components. The following conclusions are drawn:

(1) Effective drag reduction is achieved when the dimensionless groove height (*h^+^*) and width (*s^+^*) range from 8.50 to 29.75. Within this parametric regime, the V-shaped groove surface significantly modifies the near-wall turbulent structures. The maximum drag reduction of 12.33% is obtained at *h^+^* = *s^+^* = 25.29 and an inflow velocity of 80.7 m/s.

(2) The response surface analysis reveals that the interaction effects among groove height, groove width, and inflow velocity are nontrivial. The optimal groove dimensions exhibit a dependence on the inflow velocity. Under low-velocity conditions, relatively larger groove dimensions are favorable for drag reduction. In contrast, under medium-to-high flow velocities, smaller groove dimensions are required. As the inflow velocity increases from 5 m/s to 100 m/s, the optimal microstructural dimensions of the V-shaped grooves show a decreasing trend. This velocity-dependent scaling provides a quantitative design guideline for practical applications.

(3) The drag reduction mechanism is attributed to the modulation of turbulent coherent structures in the near-wall region. The V-shaped grooves suppress the streamwise, normal, and spanwise pulsating velocities, with the most pronounced suppression observed in the streamwise component. The peak values of $\left\langle u^{’}u^{’}\right\rangle$ and $\left\langle w^{’}w^{’}\right\rangle$ in the viscous near-wall region are substantially reduced, and the locations of peak values shift away from the wall.

## Figures and Tables

**Table 1:** Factor levels and codes for a second-order orthogonal rotation design.

$x_{j}$	*X*_1_ *h* (mm)	*X*_2_ *s* (mm)	*X*_3_ *U*(m/s) (${Re}_{\tau }$)
$+1.682\;\left(X_{0j}+r\Delta_{j}\right)$	0.300	0.300	100 (695)
$+1\;\left(X_{2j}\right)$	0.249	0.249	80.7 (599)
$0\;\left(X_{0j}\right)$	0.175	0.175	52.5 (443)
−1$\;\left(X_{1j}\right)$	0.101	0.101	24.3 (258)
−$1.682\;\left(X_{0j}\;-\;r\Delta_{j}\right)$	0.050	0.050	5 (85)
$\Delta_{j}=\frac{X_{2j}-X_{1j}}{2}$	0.074	0.074	28.2
$x_{j}=\frac{X_{j}\;-X_{0j}}{\Delta_{j}}$	$x_{1}=\frac{X_{1}-0.175}{0.074}$	$x_{2}=\frac{X_{2}-0.175}{0.074}$	$x_{3}=\frac{X_{3}-52.5}{28.2}$

**Table 2:** The dimensionless mesh parameters.

1	$x^{+}$			7.72
2	$y^{+}$	inside the V-shaped groove wall	${y_{v}^{+}}_{\min}$	0.78
			${y_{v}^{+}}_{\max}$	0.99
		smooth wall	${y^{+}}_{\min}$	0.99
3	$z^{+}$			1.64

**Table 3:** Comparison of Theoretical and Simulated Values.

Reτ	Theoretical Value	Simulated Value	Relative Error (%)
252	0.007227	0.007389	2.19
591	0.005801	0.005912	1.87
695	0.005238	0.005319	1.52

**Table 4:** The peak values of total TKE (m^2^/s^2^).

Smooth Wall	*y^+^*	The Peak Values of Total TKE (m^2^/s^2^)	V-Shaped Groove Wall	*y^+^*	The Peak Values of Total TKE (m^2^/s^2^)
*x* = 0.01 m	72.15	305	*x* = 0.01 m	73.23	350
*x* = 0.02 m	15.34	155	*x* = 0.02 m	15.26	171
*x* = 0.03 m	16.26	139	*x* = 0.03 m	10.14	147
*x* = 0.04 m	9.19	128	*x* = 0.04 m	9.16	124
*x* = 0.05 m	9.07	126	*x* = 0.05 m	9.02	126
*x* = 0.06 m	8.96	127	*x* = 0.06 m	8.90	124
*x* = 0.07 m	8.90	125	*x* = 0.07 m	8.84	120
*x* = 0.08 m	8.85	123	*x* = 0.08 m	8.79	119

**Table 5:** The drag reduction rate of the V-shaped groove wall.

Group	*h^+^*	*s^+^*	*U* (m/s)	${Re}_{\tau }$	*DR* (%)
1	25.29	25.29	80.7	599	12.33
2	21.16	8.58	24.3	258	7.81
3	8.58	8.58	24.3	258	6.15
4	21.16	21.16	24.3	258	3.60
5	29.75	8.50	52.5	443	3.21
6	8.50	29.75	52.5	443	2.17
7	8.58	21.16	24.3	258	1.85
8	29.75	29.75	52.5	443	0.72
9	3.58	3.58	5	85	−2.39
10	62.34	25.29	80.7	599	−5.20
11	51	29.75	52.5	443	−10.00
12	29.75	51	52.5	443	−11.49
13	25.29	62.34	80.7	599	−12.5
14	53.14	53.14	100	695	−20.25
15	62.34	62.34	80.7	599	−27.47

**Table 6:** Comparison with previous research results.

Group	*Reference*	*Optimal s^+^*	*Optimal h^+^*	${Re}_{\tau }$	*DR* (%)
1	Bechert [10]	17	8.4	low *Re_τ_*	9.01
2	Choi [13]	20	13	76	3
3	Wong [18]	17.3	8.6	395	8.7
4	Walsh [25]	12	12	<200	8.0
5	García-Mayoral [26]	15	7.5	200	8.0
6	This Work	25.29	25.29	599	12.33

## Data Availability

The original contributions presented in this study are included in the article material. Further inquiries can be directed to the corresponding author.
